# NaDES-Based Extracts by Microwave Activation from *Laurus nobilis* L. Leaves: Sustainable Multifunctional Ingredients for Potential Cosmetic and Pharmaceutical Applications

**DOI:** 10.3390/molecules30143006

**Published:** 2025-07-17

**Authors:** Debora Caviglia, Eleonora Russo, Anna Maria Schito, Francesco Saverio Robustelli della Cuna, Elena Grignani, Nicola Lionetti, Carla Villa

**Affiliations:** 1 Department of Pharmacy (DIFAR), Section of Drug and Cosmetic Chemistry, University of Genoa, 16132 Genoa, Italy; debora.caviglia@edu.unige.it (D.C.); eleonora.russo@unige.it (E.R.); 2 Department of Surgical Sciences and Integrated Diagnostics (DISC), University of Genoa, 16132 Genoa, Italy; anna.maria.schito@unige.it; 3 Environmental Research Center, Istituti Clinici Scientifici Maugeri IRCCS, 27100 Pavia, Italy; saverio.robustelli@icsmaugeri.it (F.S.R.d.C.); elena.grignani@icsmaugeri.it (E.G.); 4 Labanalysis Life Science, 20125 Milano, Italy; nicola.lionetti@labanalysis.it

**Keywords:** NaDES, ready-to-use ingredients, *Laurus nobilis* L., microwave extraction

## Abstract

*Laurus nobilis* L. is a widely cultivated plant, used for ornamental purposes, as a high-value spice crop, and in the flavor and fragrance industry. In natural medicine, it is well-known for its many beneficial properties (due to a broad spectrum of biologically active compounds) and used for the treatment of different disorders. In this study, natural deep eutectic solvents (NaDESs), coupled with microwave activation, were studied and applied for a green extraction of *L. nobilis* leaves. The main objective was to obtain a sustainable and multifunctional cosmetic and pharmaceutical ingredient (the NaDES-based extract itself), exploiting both the intrinsic cosmetic functionalities of NaDES components and the biological properties of laurel bioactive compounds. The most promising candidate was obtained from a eutectic system containing betaine, glycerol, and lactic acid. The evaluation of this NaDES-based complex reveals a considerable number of phenolic compounds (around 11.57 mg of gallic acid equivalents for a gram of fresh leaves) and a notable antioxidant activity (80.1% with respect to Trolox), with values quite constant over a period of six months. The complex exhibits effective antimicrobial activity against different Gram-positive (*S. aureus* and *S. epidermidis*) and Gram-negative (*E. coli* and *P. aeruginosa*) bacterial strains, with concentrations ranging from 3.8 to 7.5 mg/mL. Furthermore, the extract presents a pleasant fragrance, attributable to the selective extraction of different volatile aromatic compounds, as confirmed by GC-MS analysis.

## 1. Introduction

In recent years, scientific research has shifted its focus toward the design and development of green processes and products that could meet market expectations while reducing the industrial environmental impact. From the perspective of extraction, the primary objectives undoubtedly encompass the reduction and replacement of hazardous solvents, the saving of energy and resources, and the achievement of safe products of high quality, according to the principles of green extraction, as posited by Chemat et al. [1].

Even in the cosmetic and pharmaceutical fields, the investigation of new green extraction technologies from natural and renewable sources has become a predominant topic [2,3,4,5,6,7]. It is well known that plants are an endless resource of useful active compounds (i.e., antioxidant, anti-inflammatory, and antimicrobial agents) whose extraction is not always easy and environmentally friendly.

Bay laurel (*Laurus nobilis* L.) is an ancient evergreen tree belonging to the *Lauraceae* family, native to the Mediterranean region. It is widely cultivated not only as an ornamental plant but also as a high-value spice crop and as a source of ingredients for flavoring and fragrance applications [8]. Its essential oil (EO) is commonly used in folk natural remedies due to its antioxidant, antimicrobial, and anti-inflammatory properties [9,10]. Traditionally, aqueous extracts from laurel fruits and leaves were employed in natural medicine for the treatment of gastrointestinal, neurological, and urological disorders and as astringent agents in dermatological applications [11,12]. Given their wide range of properties and applications, laurel derivatives can be used in modern cosmetics and as natural complements to medicinal therapies [13,14]. For instance, it has been successfully included in anti-dandruff products and formulations for psoriasis treatment [15].

The biological properties of *L. nobilis* are attributed to a broad spectrum of biologically active compounds (BAs), including terpenoids, norisoprenoids, alkaloids, tannins, carotenoids, tocopherols, and a vast number of phenols and polyphenols, such as phenolic acids, flavonoids, and lignans [9]. They are traditionally obtained by steam distillation (for EO isolation) and by other extractive procedures with organic or hydroalcoholic solvents. Alternative approaches have recently been used to improve the release of these components from the plant matrix [11,16,17].

In the realm of extraction strategies, conventional organic solvents are commonly used for extracting aromas, perfumes, bioactive compounds, and dyes from plants, but they are often not more sustainable due to their harmfulness, high environmental impact, and flammability. In this context, NaDESs (natural deep eutectic solvents) represent a novel class of green solvents that have garnered significant attention within the scientific community. This great interest is attributable to their distinctive characteristics and versatility of application. NaDESs can be defined as supramolecular structures with very low melting points (often in a liquid state at room temperature), mainly constituted by a mixture of naturally occurring hydrogen bond donors (HBDs) and hydrogen bond acceptors (HBAs) [18].

In the natural world, a variety of plant metabolites have been identified as sources and constituents of NaDESs. In plants, the combination of these metabolites (natural eutectic mixtures) seems to fulfill several biological functions, including the capacity to facilitate the transportation of water-insoluble compounds into cellular environments, thereby contributing to the co-occurrence of hydrophilic and lipophilic compounds within the botanical matrix [19]. Synthetic NaDESs have demonstrated the capacity to replicate this inherent behavior, setting themselves up as excellent solvent candidates in diverse domains, including cosmetics and pharmaceuticals. They can be easily prepared from sugars, alcohols, organic acids, amino acids, or amines that naturally occur during plant metabolism or from eukaryotic cellular systems [20]. Their preparation is straightforward and marked by elevated purity and the absence of waste generation, aligning with the core tenets of green chemistry and green extraction [21,22]. Notably, NaDESs have emerged as promising solutions for the extraction of active principles from natural sources, marking a significant advancement in the field. In comparison to conventional solvents, these eutectic systems offer natural origin, affordability, biodegradability, sustainability, and preparation simplicity [23].

In terms of safety, NaDESs are widely regarded as harmless to both humans and the environment, referring to the toxicity data of the individual components, mainly GRAS (generally recognized as safe) compounds [24]. However, a number of eutectic mixtures studied have been demonstrated to possess a degree of toxicity when compared with the respective components, probably due to synergistic effects between the individual elements [25]. Nevertheless, a shared concern in the field of natural ingredients, particularly those used in cosmetics and pharmaceuticals, is the high solvent capacity of eutectics, which could co-extract toxic trace elements from the botanical matrix, being absorbed from a polluted environment. Recent studies on this topic [26,27] suggest that a correct adjustment for the ideal eutectic combination, adapting it to the different requirements, makes it possible to avoid this drawback.

NaDESs exhibit a high degree of versatility in extractive procedures, encompassing a broad spectrum of polarities, exceptional solubilization ability, and selectivity for various compounds. Moreover, they often improve the stability and storage of the extracted compounds of interest [28,29]. NaDESs’ polarity renders them especially well-suited for coupling with microwave (MW) irradiation, thereby enhancing the sustainability and efficiency of MW extraction processes [30].

The aforementioned properties make them very suitable for various requirements, particularly attractive for cosmetic and pharmaceutical applications [31]. Most of NaDES constituents are characterized by an intrinsic cosmetic or pharmaceutical activity and are well-known and frequently employed ingredients (i.e., organic acids, sugars, alcohols and polyols, amino acids, and quaternary ammonium salts). This last characteristic allows NaDES extracts to be directly used in the final products, avoiding costly purification steps and increasing the naturalness and performance of the compositions [32,33].

Taking all these aspects into consideration, the purpose of this work was to investigate the potential of NaDESs’ microwave-assisted extraction [34] on *L. nobilis*, applying a previously published procedure [29]. This approach aims to exploit the intrinsic cosmetic properties of the eutectic solvent together with those of laurel bioactive compounds (BAs) to obtain a natural, ready-to-use ingredient with synergistic antioxidant activity, antimicrobial functionalities, and olfactive properties.

NaDESs were prepared under microwave activation [35], combining food-grade hydrogen bond acceptors (choline chloride or betaine) and donors (lactic acid and glycerol), then used for laurel leaf extraction. To evaluate extraction efficiency and sample functionality, a preliminary screening was performed on the different NaDES-based samples in terms of total phenolic content (TPC, by Folin–Ciocalteu assay), radical scavenging activity (RSA by DPPH test), and a preliminary screening of the antimicrobial activity (minimum inhibitory concentration—MIC) on different Gram-positive and Gram-negative bacterial clinical strains.

As in previous work [28,29], for a preliminary evaluation of the stabilizing effect of NaDESs on active metabolites, the phenolic content and the antioxidant activity of the best-performing sample were monitored after 180 days of storage at room temperature for a possible decrease in TPC and DPPH values. Moreover, the volatile fraction of the extract, characterized by a unique and intense aromatic profile, was analyzed by gas chromatography–mass spectrometry (GC-MS) [36].

## 2. Results

### 2.1. MW NaDES Extraction

Following a previously published work [28], a number of natural eutectic solvents were simply prepared by microwave activation and evaluated for the extractive procedure. Four NADES-based extracts (**BGs**, **BGAs**, **CGs**, **CGAs**) were obtained from fresh laurel leaves within five minutes [24] with an easy workup, showing interesting rheological properties (low viscosity) and a distinctive olfactive profile.

The polarity of the compounds involved, in conjunction with the dielectric heating induced by microwave irradiation, led to the swift attainment and control of the set process temperature (75 °C). This occurred within a brief time frame of 10–15 s. Subsequently, the constant temperature was then maintained through power modulation within the range of 0–300 watts. A continuous feedback mechanism inherent within the MW prototype meticulously controlled this process, enabling the prevention of overheating and subsequent thermal degradation of the botanical matrix and extracts.

### 2.2. Total Phenolic Content (TPC) and Radical Scavenging Activity (RSA%)

Table 1 summarizes the main results related to the total phenolic content (TPC) and radical scavenging activity (RSA%) for all the NaDES-based extracts obtained (**BGs**, **BGAs**, **CGs**, **CGAs**). All samples demonstrated strong antioxidant activity, consistent with the corresponding TPC values.

In the evaluation of the total phenolic content, in order to exclude the possibility of interference by the eutectic mixture in the estimation of the TPC, the Folin–Ciocalteu method was slightly modified, using the eutectic extraction solvents in the blank preparation. NADES extracts exhibited total phenolic contents (TPCs) ranging from 10.12 to 11.72 mg GAE per gram of fresh leaves, with better results using betaine as the hydrogen bond acceptor. The highest TPC (11.72. ± 0.02 mg GAE/g) was recorded in the laurel extract obtained with **BG** (betaine/glycerol) NaDESs, whereas the lowest content (10.12 ± 0.03 mg GAE/g) was found in the case of **CGA** (choline/glycerol/lactic acid) solvent.

Based on the results, the extracts contained a mixture of phenolic compounds in the following order: **BGs** > **BGAs** > **CGs** > **GCAs**, with significant differences between solvents containing betaine and solvents containing choline.

This order is maintained with respect to the DPPH scavenging activities of the extracts. The **CGAs** sample is significantly different from the others and shows the lowest antimicrobial activity (62.2 ± 1.0%).

As a preliminary stability test, for compound **BGAs**, the TPC and DPPH assay values were also verified after 180 days of storage at room temperature (**BGAs** t180). The sample appears to be stable, with only a slight decrease in phenolic content (from 11.57 ± 0.02 to 11.35 ± 0.02 mg GAE/g) and not affecting its antioxidant capacity (from 80.9 ± 0.3 to 80.5 ± 0.4%).

### 2.3. Minimal Inhibitory Concentrations (MICs)

According to the guidelines of the European Committee on Antimicrobial Susceptibility Testing (EUCAST) [37], the antibacterial activity of the NaDES-based extracts (**BGs**, **BGAs**, **CGs**, **CGAs**) and the corresponding eutectic solvents (**BG**, **BGA**, **CG**, **CGA**) was evaluated against six bacterial isolates by means of the minimal inhibitory concentrations (MICs) (Table 2). They are representative of clinically relevant multidrug-resistant Gram-positive (four strains) and Gram-negative (two strains) species, including two strains of *Staphylococcus aureus* (both resistant to methicillin—MRSA), two isolates of *Staphylococcus epidermidis* (both resistant to methicillin—MRSE), one isolate of *Escherichia coli* (a strain producing class A *Klebsiella pneumoniae* carbapenemases—KPC, and resistant to carbapenems—CR), and one strain of *Pseudomonas aeruginosa* (resistant to ceftazidime–avibactam—KAZ-AVI R, and CR).

As shown in Table 2, all tested extracts showed antibacterial activity against Gram-positive strains. Only samples containing lactic acid (**CGAs** and **BGAs**) revealed good antimicrobial potential against the Gram-positive and Gram-negative clinical strains tested, with MICs ranging from 3.5 to 7.1 mg/mL.

**BGs** showed a similar activity only against Gram-positive strains (from 3.5 to 7.1 mg/mL), while **CGs** proved to have the lowest antibacterial power among the four NaDES-based samples tested, only on Gram-positive strains, mostly at concentrations of 57.7 mg/mL.

The eutectic solvents containing lactic acid (**BGA** and **GCA**) exhibited an intrinsic antimicrobial activity (from 13.5 to 27.8 mg/mL), although lower than those observed for the corresponding extracts. Neither **BG** nor **CG** showed any activity in the range of concentrations studied.

### 2.4. GC-MS Analysis

**Figure 1:**
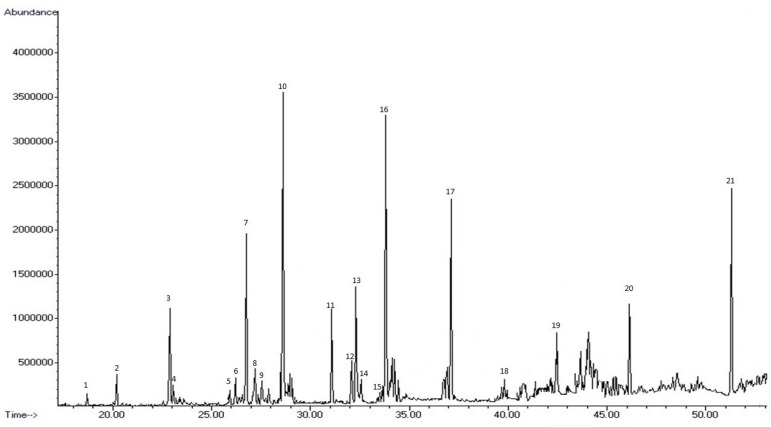
Typical GC-MS traces of **BGAs** volatile fraction. The main compounds are indicated by number. For peak identification, see Table 3.

**Figure 2:**
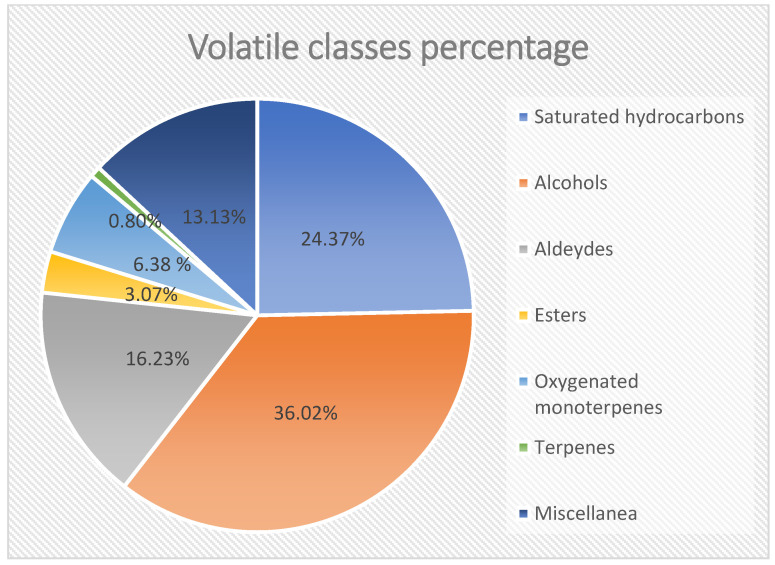
Graphical representation of the mean percentages related to the volatile compound classes, identified by GC-MS in sample **BGAs**.

The GC-MS of the volatile fraction of sample **BGAs**, selected as the most promising sample, shows the presence of 21 volatile compounds, listed in order of elution, together with their retention indices (RIs) on an Elite-5 MS column (Table 3). In Figure 1, the typical chromatogram related to **BGAs** GC-MS analysis is reported as an example. In Figure 2, percentages related to the different classes of compounds are summarized.

Major constituents were found to be alcohols (36.02%), with methyl eugenol (16.46%), 2,4-di-tert-butyl-phenol (13.27%), and eugenol (6.29%) as the most representative compounds. Saturated hydrocarbons (24.37%) represent the second largest class, with dodecane (7.15%), heptadecane (6.16%), and tridecane (4.07%). Aldehydes, accounting for 16.23% of the total volatiles, are dominated by 4-octadecanal (10.86%) and hexadecanal (5.37%). The miscellanea class (13.13%) includes only 1,3-di-tert-butylbenzene. Oxygenated monoterpenes (6.38%) are represented by linalool (4.33%), terpinen-4-ol (1.09%), and 1,8-cineole (0.96%).

## 3. Discussion

The objective of this research endeavor was to enhance the value of laurel leaves by means of a simple, environmentally friendly extraction process, obtaining a ready-to-use multifunctional ingredient.

It is noteworthy that the extraction solvents are readily available and pose minimal risk, offering a diverse array of mixtures with varying polarities, suitable for a wide range of applications. According to the previous literature [28], a number of eutectic mixtures (with different polarity) were evaluated in order to enhance the contemporary extraction of lipophilic and hydrophilic compounds with diverse functions (i.e., antioxidant hydrophilic polyphenols and hydrophobic volatile compounds with a pleasant olfactive character). In particular, the decision to select choline and betaine as the HBAs and lactic acid and glycerol (as the HBDs) is due to their proven track record in the field of NaDES extractions and for their inherent properties that can enhance the quality of the final product.

After the extraction procedure, it is of significant interest to be able to analyze and employ the sample directly after a simple mechanical filtration (to remove the spent matrix) without further purifications or work-up. Consequently, the ensuing steps facilitate the testing of the extract sample, as if it were the ultimate ingredient, which can be directly added to the final product.

The evaluation of the NaDES-based extracts in terms of phenolic amount needs to take into consideration that some components of NaDESs could potentially interfere with the measurement, particularly when using the Folin–Ciocalteu (FC) assay [32]. This interference can lead to an overestimation of the phenolic content, the extent of which cannot be predicted because it depends on the assay conditions and on the presence of other compounds. For this reason, NADES-based blanks were prepared and used in the analysis to account for any potential absorbance changes introduced by the NADES itself. This aspect has not been considered in the DPPH analysis, as the RSA of the extract–solvent complex was being evaluated as a potential ingredient in its entirety.

Once the extraction efficiency of all four eutectic samples was verified, in terms of both total phenolic content and radical scavenging activity, the most interesting aspect was related to the screening of the antimicrobial activity. In the literature, a number of studies have demonstrated that NaDESs can exhibit antimicrobial activity, particularly those containing organic acids. Their effectiveness varies greatly, depending on the specific NaDES components, their ratios, the water content, and the targeted microorganisms [42]. In order to exclude the possibility that the ingredient’s overall antimicrobial activity was primarily attributable to the properties of the solvents, the antimicrobial screening tests were also performed on the pure eutectic mixtures (**BG**, **BGA**, **CG**, **CGA**).

The comparison of the MICs obtained confirmed that the presence of lactic acid in the mixture enhances and amplifies the antimicrobial potential of the extract, not only on Gram-positive bacteria but also against Gram-negative strains. This effect is further corroborated by the findings obtained for the corresponding solvents, particularly in the context of betaine’s use as the HBA, although it should be noted that the observed efficacy is manifested at considerably higher concentrations (13.9–27 against 3.5–7.1 mg/mL).

According to the evaluation of the data related to TPC, DPPH, and the preliminary screening of the antimicrobial activity, the betaine-derived **BGAs** was selected as the most promising sample, presenting a very good performance and a pleasant fragrance too. In light of this evidence and with the prospect of utilizing this extract as a ready-to-use ingredient, a preliminary evaluation was conducted on the long-term stability (180 days) of the extract, verifying TPC and DPPH. As reported in the literature [28,29] and from the results obtained, NaDES **BGA** seems to provide high stability to the sample during storage, preserving the phenolic content and the antioxidant activity of the extract from *L. nobilis*.

With reference to the use of choline chloride as the HBA, regardless of the results obtained, a non-negligible drawback to consider is the slight amine or “fishy” odor that choline chloride imparts to both the eutectic solvent (**CG** and **CGA**) and extracts (**CGs** and **CGAs**). Moreover, considering the perspective of a potential commercial use in Europe, the utilization of this substance in the production of cosmetic products is precluded by its inclusion in the list of substances prohibited by the European Cosmetic Regulation 1223/09 (Annex II, reference n. 168). Notwithstanding, this restriction does not apply to pharmaceutical products intended for topical use. This opens the possibility for any future investigations within this scope.

The sample **BGAs** being scented, its volatile fraction by gas chromatography–mass spectrometry (GC-MS). As might be expected, the number of volatile compounds detected was considerably lower and displayed significant variation in relative percentages when compared to those observed in conventional bay laurel essential oil [10]. Nonetheless, this does not prevent the constituents from making their contribution to the sample for a pleasant and distinctive fragrance, with a combination of herbaceous, fresh, and slightly spicy notes. The aromatic contribution seems to be mainly due to the presence of linalool, eugenol, methyl eugenol, α-terpinyl acetate, and 1,8-cineole.

## 4. Materials and Methods

### 4.1. Plant Material

Fresh leaves of laurel (*L. nobilis*) were collected in the Ligurian hinterland (Alpicella, Savona, Italy, 44°23′49″ N, 8°32′41″ E) in March 2024 by a local supplier. Samples were immediately refrigerated at +4 °C and subsequently stored at −20 °C until extraction. Plants were identified according to Pignatti et al.’s method [11]. Voucher specimens (LL01, LL02) were kept in the Department of Pharmacy of the University of Genova, Italy. Distilled water was used to clean the fresh botanical material in order to remove physical impurities, then samples were chopped and stored at −20 °C until use.

### 4.2. Chemicals

Betaine (purity grade ≥ 99%), choline chloride (purity grade ≥ 99%), glycerine (purity grade ≥ 98%), lactic acid (purity grade ≥ 98%), DPPH (2,2-diphenyl-1-picrylhydrazyl) reagent, Trolox (6-hydroxy-2,5,7,8-tetramethylchroman-2-carboxylic acid) (purity grade ≥ 98%), Folin–Ciocalteu’s phenol reagent, and gallic acid (purity grade ≥ 98%) were purchased from Sigma-Aldrich (Milan, Italy).

A Milli-Q system (Millipore SA, Molsheim, France) was used to produce freshly prepared, redistilled water.

NaDESs were prepared in the laboratory according to the MW method [28] and suitably modified. NaDES abbreviations and compositions are reported as follows:

**BG** = betaine/glycerol (1:2 molar ratio);

**BGA** = betaine/glycerol/lactic acid (2:1:1 molar ratio);

**CG** = choline chloride/glycerol 1:2 (1:2 molar ratio);

**CGA** = choline chloride/glycerol/lactic acid 2:1:1 (2:1:1 molar ratio).

### 4.3. Apparatus

The MW multimode prototype (emitted power max: 900 Watt), equipped with a specially designed Pyrex reactor, a magnetron operating at 2.45 GHz, two optical fiber probes for temperature measurement, and a control unit that allows managing different process parameters such as emitted power, temperature setpoint, and mechanical stirring, was used.

A Thermoscientific UV/VIS spectrophotometer (Evolution 300, Fischer Scientific, GmbH, Schwerte, Germany) was used to record UV spectra and for RSA% and TPC analyses.

GC/MS analyses were carried out using a GC Model 6890N, coupled to a benchtop MS Agilent 5973 Network (Agilent, Santa Clara, CA, USA), using an Elite-5MS (5% phenyl methyl polysiloxane) capillary column of 30 m × 0.32 mm i.d. and a 0.32 μm thick film (Agilent, Santa Clara, CA, USA).

An Ultrasonic Cleaner Transonic 130 C.R. (ACAD Pharmaceutical Inc., Basel, Switzerland) was used.

### 4.4. Bacterial Strains

In this study, six multidrug-resistant (MDR) bacterial clinical strains (four Gram-positive and two Gram-negative) were used (see Table 2): two methicillin-resistant *Staphylococcus aureus* (MRSA), two methicillin-resistant *Staphylococcus epidermidis* (MRSE), one *Escherichia coli* resistant to carbapenems and to ceftazidime–avibactam (CAZ-AVI), and one *Pseudomonas aeruginosa* resistant to carbapenems by producing Klebsiella pneumoniae carbapenemase (KPC). All the strains were obtained from the School of Medical and Pharmaceutical Sciences (University of Genoa) and identified by VITEK^®^ 2 (Biomerieux, Firenze, Italy) or matrix-assisted laser desorption/ionization time-of-flight (MALDI-TOF) mass spectrometric technique (Biomerieux, Firenze, Italy).

### 4.5. MW NaDES Extraction

Fresh chopped leaves were added to the selected eutectic system solvents (**CG**, **CGA**, **BG**, **BGA**) in a 1:10 *w*/*w* ratio. After 5 min of sonication, the mixture was processed for 5 min, under microwave activation, at a temperature setpoint of 75 °C. After a simple filtration, the supernatant solution was kept at 4 °C until use.

### 4.6. Total Phenolic Content (TPC)

The total phenolic content of each sample was assessed by the Folin–Ciocalteu UV/VIS spectrophotometric method, using gallic acid as the reference standard [43,44].

TPCs were calculated from a calibration curve obtained from gallic acid standard solutions in concentrations ranging from 20 to 80 mg/L (R_2_ = 0.9988). Values are expressed as mg equivalents of gallic acid per gram of fresh laurel leaves (mg GAE/g). The results were derived from triplicate analyses of each extract (n = 3), normalized against a negative control of the corresponding eutectic solvent, and values are given ± standard deviation (SD).

### 4.7. Radical Scavenging Activity (RSA%)

The radical scavenging activity of every extract was measured by DPPH assay, based on the bleaching rate of the stable radical 2,2-Diphenyl-1-picrylhydrazyl (DPPH), using Trolox as the reference standard [45], obtaining a linear calibration curve ranging from 20 to 200 mg/L (R_2_ = 0.9952). A total of 0.1 mL of sample was mixed with 3.9 mL of DPPH methanolic solution (65 μM). After mixture storage for 30 min in the dark, absorbance was measured at 516 nm. The results were calculated as Trolox equivalents in solution (mg/L), and the percentage of antioxidant activity (AA%) was calculated from the ratio of decreasing absorbance of sample solution (A_0_ − A_S_) to absorbance of blank DPPH solutions (A_0_), as expressed in Equation (1) [46]. Each analysis was performed in triplicate (n = 3), and values are given ± standard deviation (SD).

AA% = (A_0_ − As)/A_0_ · 100 (1)

### 4.8. Minimal Inhibitory Concentrations (MICs)

Minimum inhibitory concentrations (MICs) were determined on six bacterial strains using the microdilution method, according to the guidelines of the European Committee on Antimicrobial Susceptibility Testing [38]. Briefly, after overnight incubation, bacteria cultures were diluted to yield a standardized inoculum of 1.5 × 10^8^ CFU/mL. Appropriate aliquots of each suspension were added to 96-well microplates containing the same volumes of serial 2-fold dilutions (ranging from 0.2 up to 110 mg/mL) of the four tested samples (**BGs**, **BGAs**, **CGs**, **CGAs**) to achieve a final bacterial concentration of about 5 × 10^5^ cells/mL. The four pure NaDESs employed (**BG**, **BGA**, **CG**, **CGA**) were also assessed at the same dilutions in order to evaluate the possible antibacterial activity of the solvents themselves. After 24 h incubation at 37 °C, the MIC values were determined as the lowest concentrations that inhibited visible bacterial growth in the wells, compared with the compound-free control, expressed in mg/mL. All tests were performed in triplicate, and MIC values were expressed as median/modal values.

### 4.9. GC/MS Analysis

NaDES samples (1 g) were diluted with 1.2 mL of ultrapure water, then extracted with *n*-hexane (3 × 1 mL). The organic phase was dried over anhydrous Na_2_SO_4_ and completely evaporated using a gentle N_2_ stream at room temperature. The residue was dissolved in 50 μL of *n*-hexane before analysis. The analyses were carried out using a GC Model 6890N, coupled to a benchtop MS Agilent 5973 Network (Agilent, Santa Clara, CA, USA). Chromatographic separation was performed using an Elite-5MS (5% phenyl methyl polysiloxane) capillary column of (30 m × 0.32 mm i.d.) and a film of 0.32 μm thickness (Agilent, San-ta Clara, CA, USA). One μL aliquot of each sample was manually injected in splitless mode [36]. The oven temperature program included an initial isotherm of 40 °C for 5 min, followed by a temperature ramp to 260 °C at 40 °C/min, and a final isotherm at this temperature for 10 min. Injector and detector temperatures were set at 250 °C and 280 °C, respectively. Mass spectra were acquired over a 40–400 amu range at 1 scan/s with an ionizing electron energy of 70 eV. The identification of the volatile compounds was performed using retention indices (RIs) and mass spectra (MS) [47], according to Adams [38], by comparison with online published data [39] and with a NIST database mass spectral library [41]. The relative amount of each component was expressed as percent peak area relative to the total peak areas from GC/MS analyses of the whole extract.

### 4.10. Statistical Analysis

Each sample was analyzed in triplicate, and Folin–Ciocalteu and DPPH data were subjected to analysis of variance (ANOVA) using GraphPad Prism version 8.0.0 for Windows, GraphPad Software, San Diego, CA, USA. Wherever F values were significant, the Bonferroni test was used for means comparison. Significance was defined at *p* < 0.001.

## 5. Conclusions

Among the emerging green procedures, natural deep eutectic solvents (NaDESs) can be considered a rapid and effective extractive approach to improve the recovery of active compounds from *L. nobilis* leaves in a sustainable way. The combination of NaDESs’ extractive properties with the biological activity of both the eutectic solvent and laurel gives rise to a synergistic performance of NaDES-based extracts.

According to the results, using a eutectic mixture of betaine, glycerol, and lactic acid in an opportune ratio, it is possible to obtain a quite stable NaDES-based extract that displays interesting properties in terms of antioxidant and antimicrobial activities and unique olfactive character. It is submitted that, when considered as a whole, these characteristics, after suitable in-depth studies, could allow this extract to be evaluated for its potential use as a ready-to-use multifunctional ingredient for cosmetic and pharmaceutical applications.

The use of these eutectic systems, as an alternative to organic solvents, can also align with the market trend towards natural products and fulfill the needs for functionality and skin efficacy in dermocosmetic formulations.

## Figures and Tables

**Table 1:** Total phenolic content (TPC) and radical scavenging activity (RSA%) of NaDES samples (**BGs**, **BGAs**, **CGs**, **CGAs**) by means of DPPH (2,2-Diphenyl-1-picrylhydrazyl) and Folin–Ciocalteu assays (mean value ± standard deviation SD) of three independent experiments (n = 3). Numbers followed by different letters within the same column indicate significant differences among results (*p* < 0.001, Bonferroni test).

NaDES Sample *	TPC (mg GAE/g ± SD)	RSA (% ± SD)
**BGs**	11.72 ± 0.02 ^a^	88.1 ± 0.1 ^a^
**BGAs**	11.57 ± 0.02 ^a^	80.9 ± 0.3 ^b^
**CGs**	10.38 ± 0.07 ^b^	77.8 ± 0.4 ^b^
**CGAs**	10.12 ± 0.03 ^b^	62.2 ± 1.0 ^c^

* mg GAE/g = mg equivalents of gallic acid for g of fresh laurel leaves.

**Table 2:** Minimal inhibitory concentrations (MICs) expressed as mg/mL of NaDESs (**BG, BGA, CG, CGA**) and NaDES-based samples (**BGs**, **BGAs**, **CGs**, **CGAs**) against Gram-positive and Gram-negative bacterial species. Experiments were carried out in triplicate. The degree of concordance in all the experiments was 3/3. Variation among triplicate samples was less than 10%.

		MICs (mg/mL)							
Bacterial Species	Strain	BG *	BGs	BGA	BGAs	CG *	CGs	CGA	CGAs
*S. aureus* (MRSA)	17	na	7.0	27.8	7.1	na	55.7	27.1	7.0
	18	na	3.5	27.8	3.5	na	55.7	27.1	3.5
*S. epidermidis* (MRSE)	2	na	3.5	27.8	3.5	na	55.7	27.1	3.5
	22	na	3.5	27.8	3.5	na	27.9	27.1	3.5
*E. coli* (CR)	477	na	na	13.9	3.5	na	na	13.5	3.5
*P. aeruginosa* (CR, K)	259	na	na	13.9	3.5	na	na	13.5	3.5

* na = no activity detected in the range of concentrations studied (from 110 to 0.2 mg/mL); MRSA = methicillin-resistant *Staphylococcus aureus*; MRSE = methicillin-resistant *Staphylococcus epidermidis*; CR = carbapenemase-resistant; K = strain producing class A *Klebsiella pneumoniae*.

**Table 3:** GC-MS analysis of **BGAs** volatile fraction: retention indices, relative percentage, identification method, and olfactive description of each identified compound in three different analytical samples (**BGAs_1_, BGAs_2_, BGAs_3_**).

Compound ^a^		RI ^b^	RI ^c^	Relative %			Mean % (± SD)	Identif. Method ^d^	Olfactive Description
				BGAs_1_	BGAs_2_	BGAs_3_			
1	Decane	1000	1000	0.48	-	0.58	0.53 ± 0.08	STD	alkane
2	1.8-cineole	1042	1043	1.34	0.50	1.06	0.96 ± 0.43	NIST, RI	mint, sweety
3	Linalool	1106	1109	5.60	3.72	3.67	4.33 ± 1.10	NIST, RI	flower, lavender
4	2,4-dimethyldecane	1115	1113	0.90	0.42	0.62	0.65 ± 0.24	NIST, RI	alkane
5	Borneol	1188	1186	0.73	0.90	-	0.81 ± 0.12	NIST, RI	camphor
6	Terpinen-4-ol	1192	1194	1.32	1.31	0.64	1.09 ± 0.39	NIST, RI	turpentine, nutmeg
7	Dodecane	1200	1200	8.28	5.70	7.47	7.15 ± 1.32	STD	alkane
8	4,8-dimethyl-undecane	1212	1221	2.30	1.43	2.49	2.08 ± 0.57	NIST, RI	alkane
9	2,4-dimethylundecane	1223	1230	1.60	0.66	1.31	1.19 ± 0.48	NIST, RI	alkane
10	1,3-di-tert-butylbenzene	1249	1259	15.77	9.17	14.44	13.13 ± 3.49	NIST, RI	-
11	Tridecane	1300	1330	4.06	3.13	5.02	4.07 ± 0.95	STD	alkane
12	α-terpinyl acetate	1356	1359	2.37	4.28	2.64	3.1 ± 1.03	NIST, RI	wax
13	Eugenol	1363	1365	6.44	9.04	3.38	6.29 ± 2.83	NIST, RI	clove, honey
14	2-methyl-tridecane	1366	1373	1.24	1.17	1.55	1.32 ± 0.20	NIST, RI	alkane
15	Tetradecane	1400	1400	0.41	0.48	0.69	0.53 ± 0.14	NIST, RI	alkane
16	Methyl eugenol	1408	1410	15.70	19.56	14.13	16.46 ± 2.80	NIST, RI	clove, spice
17	2,4-di-tert-butyl-phenol	1513	1517	10.97	13.70	15.14	13.27 ± 2.12	NIST, RI	-
18	Hexadecane	1600	1600	1.08	0.92	1.32	1.11 ± 0.20	STD	alkane
19	Heptadecane	1700	1700	5.31	5.86	7.31	6.16 ± 1.04	STD	alkane
20	Hexadecanal	1831	1830	4.57	6.18	5.36	5.37 ± 0.80	NIST, RI	alkane
21	Octadecanal	2037	2036	9.53	11.89	11.16	10.86 ± 1.21	NIST, RI	oil

^a^ Compounds listed in order of their elution on an Elite-5 column. ^b^ RI = retention indices according to Adams [38] and with online published data [39]. ^c^ RI = retention indices determined on an Elite-5 column using a homologous series of n-hydrocarbons [40]. ^d^ Method of identification: STD = standard pure compound; MS = mass spectrum; NIST = National Institute of Standards and Technology database library [41]; RI = retention indices in agreement with the literature values [38,39].

## Data Availability

Data are available on request from the corresponding author.

## Abbreviations

The following abbreviations are used in this manuscript:

NaDES	Natural deep eutectic solvent
BG	Betaine/glycerol NaDES
BGA	Betaine/glycerol/lactic acid NaDES
CG	Choline/glycerol NaDES
CGA	Choline/glycerol/lactic acid NaDES
BGs	Betaine/glycerol NaDES-based extract sample
BGAs	Betaine/glycerol/lactic acid NaDES-based extract sample
CGs	Choline/glycerol NaDES-based extract sample
CGAs	Choline/glycerol/lactic acid NaDES-based extract sample
MWs	Microwaves
MICs	Minimal inhibitory concentrations
NIST	National Institute of Standards and Technology
RIs	Retention indices
STD	GC-MS standard
GAEs	Gallic acid equivalents
